# React and act: a qualitative study of how nursing home leaders follow up on staff-to-resident abuse

**DOI:** 10.1186/s12913-020-05969-x

**Published:** 2020-12-01

**Authors:** Janne Myhre, Susan Saga, Wenche Malmedal, Joan Ostaszkiewicz, Sigrid Nakrem

**Affiliations:** 1 Department of Public Health and Nursing, Faculty of Medicine and Health Sciences NTNU, Norwegian University of Science and Technology, Trondheim, Norway; 2 Centre for Quality and Patient Safety Research – Barwon Health Partnership, Institute for Healthcare Transformation, Deakin University, Geelong, VIC Australia; 3 National Ageing Research Institute, Parkville, VIC 3052 Australia

**Keywords:** Nursing home, Leaders, Patient safety, Organisational learning, Elder abuse, Staff-to-resident abuse, Adverse events

## Abstract

**Background:**

Elder abuse in nursing homes is a complex multifactorial problem and entails various associations across personal, social, and organisational factors. One way leaders can prevent abuse and promote quality and safety for residents is to follow up on any problems that may arise in clinical practice in a way that facilitates learning. How nursing home leaders follow up and what they follow up on might reflect their perceptions of abuse, its causal factors, and the prevention strategies used in the nursing home. The aim of this study was to explore how nursing home leaders follow up on reports and information regarding staff-to-resident abuse.

**Methods:**

A qualitative explorative design was used. The sample comprised 43 participants from two levels of nursing home leadership representing six municipalities and 21 nursing homes in Norway. Focus group interviews were conducted with 28 care managers, and individual interviews took place with 15 nursing home directors. The constant comparative method was used for the analyses.

**Results:**

Nursing home leaders followed up incidents of staff-to-resident abuse on three different levels as follows: 1) on an individual level, leaders performed investigations and meetings, guidance, supervision, and occasionally relocated staff members; 2) on a group level, feedback, openness, and reflection for shared understanding were strategies leaders used; and 3) on an organisational level, the main solutions were to adjust to available resources, training, and education. We found that leaders had difficulties defining harm and a perceived lack of power to follow up on all levels. In addition, they did not have adequate tools for evaluating the effect of the measures that were taken.

**Conclusions:**

Nursing home leaders need to be clear about how they should follow up incidents of elder abuse on different levels in the organisation and about their role in preventing elder abuse. Evaluation tools that facilitate systematic organisational learning are needed. Nursing homes must operate as open, blame-free cultures that acknowledge that incidents of elder abuse in patient care arise not only from the actions of individuals but also from the complex everyday life of which they are a part and in which they operate.

## Background

Nursing homes are institutions, with a dual demand of serving as a home for residents [1] but also providing social services and complex health care day-and-night [2]. Residents in these institutions have chronic diseases, complex care needs, and physical impairments, and they may have dementia or other forms of cognitive impairment [3]. Many residents have significant neuropsychiatric symptoms such as agitation, aggression, anxiety, depression, apathy, and psychosis [4]. In addition to the complexity of residents’ needs, complexity exists in that nursing homes consist of different stakeholders such as staff, leaders, relatives, and residents themselves in constantly shifting interactions [5, 6]. Patient safety and quality of care in nursing homes is multifactorial, comprising associations between organisational factors, the technical performance of care, and the organisation’s culture [2, 7] as well as values, attitudes, and knowledge in society and its policies [8]. Leaders of these institutions have a responsibility to ensure that residents’ human rights are protected, and that residents are safe and free from harm [9, 10]. However, research have found high rates of staff-to-resident abuse in nursing homes [11–13].

The World Health Organisation (WHO) defines elder abuse as ‘a single, or repeated act, or lack of appropriate action, occurring within any relationship where there is an expectation of trust which cause harm or distress to an older person ( [14], p 3). Abuse can be subdivided into physical abuse (e.g., slapping, pushing, inappropriate use of restraints), emotional or psychological abuse (e.g., humiliating, threatening, or treating a resident like a child), financial or material abuse (e.g., misusing power of attorney, stealing, selling personal belongings without consent), sexual abuse (e.g., any unwanted sexual activity), and neglect (e.g., failing to provide for basic health or medical needs, abandonment) [15]. For nursing home residents, the consequences of abuse include reduced quality of life, psychological and physical harm, loss of assets, and increased morbidity and mortality [16].

A survey of nursing home staff in Norway found that 60.3% had exposed a resident to one or more incidents of abuse in the past year [13]. The majority of staff in this study reported that they had never committed financial or sexual abuse against a resident. Physical abuse was reported by 9.6%, and psychological abuse and neglect had the highest prevalence, with 40.5 and 46.9% respectively [13]. A meta-analysis of the prevalence of elder abuse in nursing homes estimated a pooled prevalence of 64.2% of staff-to-resident abuse in the past year; additionally, in this study, psychological abuse and neglect had the highest prevalence [11]. Psychological abuse and neglect are often related to care activities such as leaving a resident alone [12], omitting to change wet incontinence pads [17], ignoring or rejecting residents [12, 13], omitting to provide oral health care [13], and arguing with or shouting at residents [12, 13].

The many different interpretations on what constitutes abuse and its severity complicate its detection, reporting, and management in nursing homes [9, 18, 19]. In addition, in nursing homes, elder abuse has been conceptualised as a specific form of abuse, such as institutional abuse [20] and a context where abuse and neglect take place [21]. The relationship between staff and residents is characterized by differences in power, and the resident is dependent on staff to fulfil most of his/her needs [22, 23]. But also due to the fact that regulations and rules within the institutional context can be abusive itself, such as deny residents choices in everyday life, e.g. when to dress and undress and have meals, and sharing room and space with other residents. Risk factors for abuse within institutions are also complex multifactorial problems entailing various associations between personal, social, and organisational factors [24, 25]. This means that the risk of some forms of staff-to-resident abuse extends beyond the traits and circumstances of the older adults and the staff who abuse them [25]. At the same time, intentionally criminal abuse should be reported to the police. In order to do so, openness in the organisation is crucial so that the nursing home leaders get knowledge about the situation that has occurred or the suspicion of what is occurring.

Preventing harm is a core principle of health care and a responsibility of leaders [26]. Leadership is critical for patient safety and prevention of harm since leaders influence the culture and care practices in nursing homes and set policies for staff [27]. Good leadership is an essential factor in developing staff’s understanding of residents’ needs [28, 29]. According to the Institute for Healthcare Improvement (IHI) report “Leading a Culture of Safety: A Blueprint for Success” [26], healthcare organizations that are successful in improving safety and eliminating harm have leaders who understand and commit to the principles of a ‘just culture’. The IHI defines a ‘just culture’ as one that focuses on identifying and correcting system factors without blaming individuals for human mistakes and, at the same time, establishing zero tolerance for reckless behaviour [26]. In order to do so, leaders need to investigate each event to determine whether the incident was caused by human error (e.g., slips), at-risk behaviour (e.g., taking shortcuts), or reckless behaviour (e.g., ignoring required safety steps). The result of the investigation should determine the response and the follow-up [26].

To be able to effectively investigate and follow up, leaders need comprehensive information about the care and service provided. This information can be obtained from the formal reporting systems or from informal reports, such as verbal information and observation. The use of a reporting system and various information sources is linked to the belief that patient safety can be improved by learning from incidents and ‘near misses’ [30]. Learning can take place at the individual and at the organisational level. Individual learning focuses on increasing knowledge and skills for individual staff members to enable them to do a better job, while organisational learning involves sharing the thoughts and actions of all the individuals in the organisation; furthermore, organisational learning entails a cultural change [30, 31]. Organisational learning is mediated through individual learning or problem-solving processes but the opposite is not true [32]. Argyis and Schön divided organisational learning into ‘single-loop learning’, which refers to the correction of errors without significantly changing the overall safety culture, and ‘double-loop learning’, which refers to a cultural change that involves questioning and alterations of the governing values within the organisation [31].

In Norway, the responsibility of nursing home leaders to follow up on information and adverse-event reports is formally regulated in the national regulation of management and quality improvement in healthcare services [33]. This regulation points to leaders’ responsibilities to monitor the overall quality and safety of resident care and to establish a culture of openness where events are reported, openly discussed, and analysed. The follow-up for incidents involves analysing the causes and implementing preventive measures designed to ensure that incidents do not happen again. Any follow-up should also include an evaluation of the measures taken in response to an incident [33]. Knowledge and understanding of how nursing home leaders follow up on information and reports regarding elder abuse are essential because one might assume that their reactions and responses reflect their perceptions of abuse, its causal factors, and the prevention strategies applied in the nursing home. To the best of our knowledge, this is the first study to investigate how nursing home leaders follow up on information and reports of staff-to-resident abuse.

### Aim of the study

This study aimed to explore how nursing home leaders follow up on reports and information regarding staff-to-resident abuse.

## Methods

### Design

The present study is part of a larger study funded by the Research Council of Norway (NFR); project number 262697. In the present study we used a qualitative exploratory design that included both focus group and individual interviews. Results from the first analyses of the focus group interviews have been published previously [19], and selected data related to how the lower level of managers understand the consept elder abuse was analysed. However, we did not report from data about follow-up on elder abuse. This is also reflected in the interview guide. The present study reports unpublished data from the focus groups in addition to new data from individual interviews with nursing home directors. By combining individual interviews with nursing home directors and focus group interviews with care managers, the present study also compares and contrasts leadership levels.

In Norway, all nursing homes have two levels of leadership: the nursing home director and the nursing home leader team. These two leader levels can influence each other through their hierarchical relationship, and together they can influence the quality of care and patient safety [27]. By gathering information from the perspectives of both levels, the intention of the present work was to develop a deeper understanding of how nursing home leaders follow up on staff-to-resident abuse in nursing homes. This study follows the Consolidated Criteria for Reporting Qualitative Research (COREQ) [34].

### Settings

The vast majority of Norwegian nursing homes are owned and run by municipalities, financed by taxes and resident payment; less than 10% are private non-profit institutions [35]. Nursing homes are led by nursing home directors, who are administrative managers for entire facilities, and some nursing home directors are administrators for more than one nursing home. The next level of managers is a leadership team comprising ward leaders, a quality leader, and, in some municipalities, a service leader [36]. The ward leader is a registered nurse (RN) who is responsible for staff and residents and the budget for his or her ward. The quality leader is an RN who monitors the overall quality of care in collaboration with ward leaders. The service leader is responsible for service staff such as activity coordinators, cleaning staff and kitchen staff and budgets related to these staff. Individuals on the leader team provide the closest leadership to staff and residents, but they do not provide direct hands-on care.

The provision of care in Norwegian nursing homes is delivered under the “National Regulation of Quality of Care” [37]. This regulation aims to ensure that residents’ basic needs are met, including their psychological and physical needs, and that their dignity, autonomy and self-respect are preserved. Health personnel have a responsibility to report any adverse event that may endanger patient safety. This is formally regulated in the “National Health Personnel Act” [38]. Abuse can be classified within the category of patient safety and adverse events that health personnel are responsible for reporting. In the present study, we used the term adverse event to refer to events and incidents of intentional or unintentional abuse where the outcome for the resident is harmful or potentially harmful. This term also includes failure to deliver needed care, defined as the omission or neglect of delivering any aspect of required resident care.

### Sample

The study sample was recruited from 21 nursing homes in six municipalities in Norway.

These six municipalities can be divided into one small municipality with population size < 4999, two middle range municipalities with 5000–19,999 inhabitants and three large municipalities with population size > 20,000. Inclusion criteria were as follows: a) the person is employed in a leadership position in a nursing home and (b) is employed full-time in that role. We recruited municipalities and nursing home leaders with a step-wise approach, because we wanted to get a theoretical sampling until saturation of data was achieved [39, 40]. In all, 43 participants were recruited: 15 individual interviews were conducted with nursing home directors and 6 focus group interviews were conducted with a total of 28 participants comprising twenty-three ward leaders, two quality leaders and three service leaders. In this study, we chose to refer to all 28 participants in the six focus group interviews as ‘care managers’ since all were members of the leadership team. The characteristics of the participants are presented in Table 1.

**Table 1:** Demographics of the study participants (*n* = 43)

Background characteristics	Care manager (***n*** = 28) Number (%)	Nursing home director (***n*** = 15) Number (%)
**Age (years)**		
30–39	6 (22)	1 (7)
40–49	11 (39)	2 (13)
≥ 50	11 (39)	12 (80)
**Sex**		
Female	25 (89)	13 (87)
Male	3 (11)	2 (13)
**Number of beds managing:**		
0	5 (17)	
10–19	8 (29)	
20–29	8 (29)	
30–40	6 (21)	
40–59	1 (4)	8 (53)
60–99		3 (20)
100–199		3 (20)
≥ 200		1 (7)
**Number of staff managing:**		
0	2 (7)	
10–29	9 (33)	
30–49	11 (39)	
50–99	6 (21)	5 (33)
100–199		6 (40)
≥ 200		4 (27)
**Working experience in this position**		
0–4	20 (71)	8 (53)
5–9	7 (25)	3 (20)
≥ 10	1 (4)	4 (27)
**Total working experience as a leader in years**		
0–4	11 (39)	1 (7)
5–9	6 (22)	1 (7)
≥ 10	11 (39)	13 (86)
**Formal leader education**		
0	1 (4)	1 (7)
0,5–1 years course	18 (64)	5 (33)
1–2 years course	3 (11)	2 (13)
Master’s Degree	6 (21)	7 (47)

### Recruitment and data collection

The recruitment period was from August 2018 to the end of January 2019. The first recruitment e-mail was sent to healthcare managers in 11 municipalities, both urban and rural areas. Healthcare managers from six municipalities accepted the invitation, while five healthcare managers stated that nursing home leaders in their municipalities did not have time to participate. Subsequently, a second recruitment e-mail was sent to all nursing home directors in the six municipalities that had accepted the invitation. The second recruitment e-mail included two invitation letters: one letter to nursing home directors and the other for nursing home directors to forward to care managers in their nursing homes. The care managers were invited to participate in focus group interviews, while the nursing home directors were invited to participate in individual interviews since there are few nursing home directors in each municipality and it was difficult to gather them for focus group interviews.

All interviews took place in a meeting room in a nursing home in the included municipalities. Each focus group interview lasted approximately 90 min, and each individual interview lasted approximately 60 min. Before the interview started participants were asked about demographic information, see Table 1. JM was the moderator for all six focus group interviews. Co -moderator was SN in two group interviews, SS in one group interview and two researchers from the larger research team for the other three interviews All 15 individual interviews were conducted by JM. We used the same interview guide for the focus group interviews with the care managers and the individual interviews with the nursing home directors (Table 2). The researchers made the interview guide after studying the literature on elder abuse in nursing homes. Participants were asked about their experiences and thoughts on the topic of elder abuse and how they follow up on these situations. We encouraged participants to speak freely. All interviews were recorded and transcribed verbatim, retaining pauses and emotional expressions. Data from the focus group interviews exploring the care managers perceptions of elder abuse (the first topic in the interview guide) were published in a previous paper [19]. The present study includes a secondary analysis of data from the focus group interviews with the care managers and new analysis of the individual interviews with nursing home directors focusing on the second and last topic in the interview guide.

**Table 2:** Interview guide

Topic	Key questions
Introduction	Can you describe what you will define as abuse and neglect in nursing homes?
Your experiences of elder abuse and neglect	Within these situations (Fig. 1), and these categories; *physical abuse, psychological abuse, financial abuse, sexual abuse and neglect,* can you describe your experience of elder abuse and neglect?
Communication of elder abuse and neglect	Can you describe how you get knowledge about situations of elder abuse and neglect in the nursing home? What do you think are barriers and enablers to reporting elder abuse and neglect?
How to follow up on elder abuse and neglect	When you get knowledge about situations of elder abuse and neglect, how do you follow it up? What do you do to prevent it from happening again?
Closure	Do you have anything to add that has not yet been mentioned? How did you experience participating in this focus group?

Note: The results from the topic 1 ‘Experience of elder abuse ‘is published with data from focus group interviews with care managers

### Data analysis

The constant comparative method allowed us to generate a thematic understanding through an open exploration of experiences described by nursing home leaders [39, 40], and permits the possible identification of themes and differences between the two different leadership levels. The analysis started immediately after each interview, when the first author listened to the recorded interview. We used memo writing throughout the process of both data collection and analysis. The memo document worked as a file of emerging ideas, thoughts, questions and categories [39]. An open line-by-line coding of the transcribed interviews was the next step in the analysis process [39, 40]. Next, we compared codes from the open coding for commonalities and frequencies. Further, codes were then clustered to develop sub-categories. To construct the final categories and main theme, the sub-categories was examined. We went back and forth between memo writing, data analysis and contextualisation to ensure that the emerging categories and themes fitted the situations explored [39]. Comparisons between groups were conducted in three main steps: 1) comparison within a single interview; 2) comparison between interviews within the same group; and 3) comparison of interviews from different groups [39, 40]. The first and last author (JM and SN) coded all transcribed interviews independently in order to diminish research bias and increase credibility. All authors met several times during the analysis process to discuss codes, their connections and reach consensus.

### Ethical consideration

Ethical approval for this study was granted by the Norwegian Center for Research Data (NSD), Ref. no. 60322. All participants were provided with written information about the study and gave written consent to participate in the interviews and for the use of the data collected from the interviews.

## Results

**Fig. 1:**
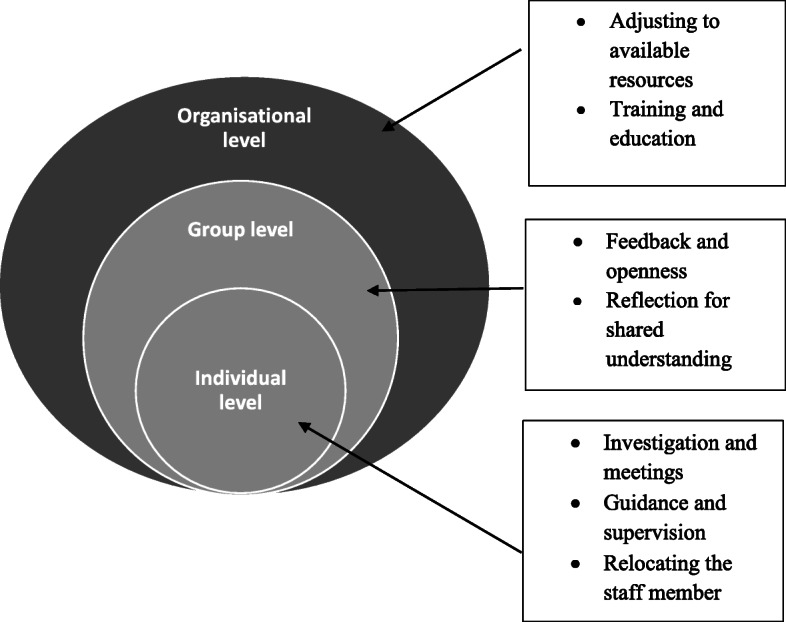
Follow-up on reports and information about abuse by nursing home leaders

In the beginning of the interviews, participants were reluctant to share their experiences of staff-to-resident abuse in their nursing home. Participants considered the term ‘abuse’ and the topic of staff-to-resident abuse, which was mainly related to intentional physical acts and sexual abuse, as highly sensitive. Few participants had experienced severe sexual or financial abuse on the part of staff. Most participants had experience mainly in regard to following up on incidents of physical abuse such as use of restraint or rough handling during care, psychological abuse and neglect. In the analyses, we found that nursing home leaders follow up on elder abuse in nursing homes on three different levels: *1) an individual level; 2) a group level; 3) and an organisational level.* An additional finding involved the differences between how nursing home directors and care managers were involved in the follow-up and how they perceived the root causes of the abuse. Their involvement and perceptions influenced how they reacted and acted upon and how they followed up on incidents. Analytical categories and sub-categories are presented in Fig. 1.

### Follow-up on an individual level

All participants described staff-to-resident abuse as related primarily to individual characteristics of certain staff members. For example, they stated that some staff members had personalities and/or attitudes that were unsuitable for working with older people in a nursing home. Other factors that were mentioned included staff’s personal problems, lack of knowledge, stress, and burnout. Both care managers and nursing home directors expressed that they did not want information or reports from staff in relation to patient abuse to be anonymous because they needed to know the name of the person to whom they should speak. “Investigation and meetings”, “guidance and supervision” and “relocating the staff member” were noted as ways the participants followed up on information and reports of incidents or potential incidents of abuse at the individual level.

#### Investigation and meetings

All participants stated that, when they received information about an incident of abuse, they invited the staff member involved in the situation to a meeting. However, participants found these meetings were ‘difficult’. In part, the difficulty was said to be related to differences in peoples’ accounts of the incident. For example, one participant explained that, often, the staff member involved in the situation had one version of what happened; the staff member who had observed the situation saw it another way; and the resident involved had a third story. Difficulty trusting the resident’s version of what happened was related to his or her cognitive status; one care manager remarked: *“What I think is difficult here is that these residents … they are almost like children. What is fiction and what is truth of what they say?”* (Care manager, group 1). All participants described being uncertain about which of the three stories they should consider to be the most accurate. The nursing home leaders said that they wanted to be sure and to have evidence before confronting or following up on information regarding abuse by a staff member. Because of this, both the care managers and the nursing home directors stated that the care managers who received the information first, were required to undertake a thorough investigation. This investigation was allegedly conducted before a meeting, but it could also take place after a meeting with the staff member involved:

*“As a leader, you must do a lot of investigation in the beginning of a case. Because when you talk to the staff member you are going to follow up, that staff member has a completely different version than the one you have been told”.* (Care manager, group 3)

Having received several written adverse-event reports related to the same staff member was perceived as evidence, as were care managers observations and accounts of the incident. However, one problem was that it could be difficult to elicit adverse-event reports related to abuse from staff. Some leaders stated that staff-to-resident abuse was too sensitive a topic to be reported in the adverse-event reporting system.

Participants indicated that care managers were responsible for most of the follow-up on an individual level. Nursing home directors were involved in the investigation of cases perceived as severe. Several participants also noted that they had to involve the human resource department in handling staff-to-resident abuse if the abuse was serious or the staff member disagreed with the finding. Only a few participants had experienced cases with clear evidence of severe physical, sexual or financial abuse. Those leaders who had experienced dealing with such incidents had contacted the police and the health authorities.

#### Guidance and supervision

In situations where abuse took the form of disrespectful behaviour toward a resident or where staff did not reflect on their own practice, participants said that they attempted to guide the staff member to reflect on and to understand the situation during a meeting. One nursing home director remarked: *“The very best is if you can talk things through during the meeting and the staff themselves reflect and realize that what they have done isn’t good practice”* (Nursing home director, 6). Another way to follow up after a meeting was to give the staff member a written reprimand. At the same time, several participants felt that a written reprimand was a serious response and an action they worked to avoid. According to several participants, an additional way of following up was to provide guidance and supervision with the intention of getting the staff member to change his or her behaviour. This could be used if they suspected that the staff member lacked technical or relational knowledge and skills. In such cases, care managers and nursing home directors stated that care managers could follow up by having conversations with the staff member, while a registered nurse could supervise the staff member in his or her daily interactions with residents. Although all participants mentioned guidance and supervision in daily care as a way to follow up on an individual level, few participants had used this strategy themselves. The main barrier to doing so was stated to be the consideration of confidentiality for the staff member in need of follow-up in relation to other staff.

#### Relocating the staff member

Both care managers and nursing home directors indicated that it was difficult to go through the process of dismissing a staff member who was considered unsuitable for working in a nursing home setting, without having enough evidence. One care manager remarked: *“You need a lot of documentation in those cases, and I feel that staff can do quite a lot and still have strong protection”* (Care manager, group 2). Participants indicated that, in situations involving complaints from residents on a ward but insufficient evidence to dismiss the staff member, he or she was moved away from the resident. According to the nursing home directors and the care managers, relocation was done on the ward away from the resident who had complained to another ward in the nursing home or to another nursing home, where the staff member continued the same care activities.

*“We have had one case here that involves rough handling of a resident. The entire human resources section was connected to that case, and it ended up with moving the staff member to another nursing home.”* (Care manager, group 4)

Other participants stated that staff members could be relegated to another role or to other tasks that did not involve direct resident care, e.g. kitchen or cleaning work, which might result in the staff members choosing to quit his or her job at the nursing home.

### Follow-up on a group level

Participants stated that caring for residents with dementia and aggressive behaviours was a daily challenge for all staff, especially residents who resisted care. Therefore, the leaders felt they had to intervene not only for individual staff members but also at a group level. Participants discussed how to define elder abuse and said that the organisational culture influenced what was perceived as acceptable staff behaviour. “Feedback and openness” and “reflection for shared understanding” were how the leaders followed up on information and reports of incidents or potential incidents of abuse on a group level.

#### Feedback and openness

Both care managers and nursing home directors suggested that it was important to build a culture of openness within the nursing home. One way of doing that was by giving feedback on reports of incidents of abuse or potential abuse at a group level. However, how this was done depended on internal routines in the different municipalities. Some participants said that they gave staff feedback on all reports of adverse events at staff meetings at the ward. Reports related to abuse were read and discussed anonymously by the staff-member group with the intention to learn; for example, incidents of physical and chemical restraint were handled this way: *“Then I raised it at a staff meeting; What do we do in such situations and which options do we have?”* (Care manager, group 2).

In addition, several stated that they sent information by e-mail to staff if they received reports concerning situations that they wanted all staff to be aware of. A few participants said that they had monthly meetings in the nursing home and meetings together with all the nursing homes in the municipality twice a year where adverse-event reports were discussed. However, according to most participants, there are no routines for handling and giving feedback on reports. Participants from these municipalities expressed that they gave feedback on reports of adverse events only to staff involved in the incidents on an individual level.

In terms of providing feedback, all participants described difficulty identifying all harmful incidents and obtaining comprehensive information about every incident of potential or actual abuse in the nursing home. To counterbalance this difficulty, both care managers and nursing home directors pointed out that they attempted to build a culture of openness that would prompt staff to report such incidents. Hence, participants emphasised the importance of reporting and said they encouraged staff to offer feedback and to guide each other during their daily work. Several also suggested that the feedback staff gave to each other was more important than the feedback they gave as leaders.

An additional finding was the difficulties participants experienced when evaluating measures taken after an adverse-event report:

*“I think it is very difficult to evaluate this. You can look at the number of adverse-event reports. But we work to get staff to write adverse-event reports, while we at the same time are putting in place measures with the intention to get fewer adverse-event reports, and then it will not be right to evaluate by just looking at the numbers. So, the evaluation is very much through behaviour changes in individual staff members, or the experience the staff and the manager have of the climate in the ward”.* (Nursing home director, 4)

This indicates that, because of the difficulties described by the participants involved in evaluating at a group level, they perceived it as easier to evaluate behaviour change in an individual staff member.

#### Reflection for shared understanding

All participants viewed reflection as a way to follow up on information and reports of abuse on a group level. Sometimes when leaders obtained second-hand information about abuse, they felt it was difficult to approach the individual staff member and, instead, attempted to address the problem on a group level:

*“It isn’t always easy for me as leader when I get information like: ‘Something happened three weeks ago, but I didn’t see it myself’; it is the resident’s experience of what the staff member has done. Then I make it a case and bring it to a reflection meeting with all the staff, and hopefully someone will take it into account”.* (Care manager, group 3)

The participants also believed that reflection could prevent abuse from occurring in the first place. They pointed out that reflection was important because of the complex, everyday care required in the nursing home and especially related to caring for residents with aggressive behaviour. Participants identified different reflection models such as the Targeted Interdisciplinary Model for Evaluation and Treatment of Neuropsychiatric Symptoms (TIME), person-centred care in dementia (VIPS), and the systematic model for ethical reflection (SME) as helpful resources for understanding residents with aggressive behaviour. The care managers also stated that participating in reflection meetings gave them insight into the nursing home culture. But even though all participants identified reflection as important, they also indicated that lack of time and resources were barriers for them to organise and participate in reflection meetings to the extent that they wished. Several care managers further stated that they did not have time to organise reflection at all:

*“We can have a conversation after something has happened and talk about that situation. But we do not have time to do planned ethical reflection”.* (Care managers, group 4)

Due to a lack of resources and difficulties organising reflection meetings, some nursing home directors referred to ethical reflection as ‘everyday reflection’ and considered it something that the staff should do on their own during the shift. One nursing home director said: *“You don’t have to feel that you need to spend extra time on reflection. It should be a natural part of the workday in the nursing home”* (Nursing home director, 15). At the same time, other participants stated that knowing whether or not reflection actually improved the culture in the nursing home was problematic.

### Follow-up on an organisational level

Both care managers and nursing home directors also linked abuse to organisational factors such as lack of staff with formal education and knowledge about caring for residents with dementia. Here, care managers and nursing home directors had different perceptions of whether inadequate staffing was a factor related to the incidence of abuse in nursing homes. The sub-categories “Adjusting to available resources” and “Training and education” emerged as ways the participants follow up on information and reports of incidents or potential incidents of abuse at an organisational level.

#### Adjusting to available resources

All care managers described lack of staff resources as a factor that increased the risk of abuse in the nursing home. At the same time, they also expressed a powerlessness related to the situation. Therefore, when abuse by staff was caused by an inadequate staffing level, it tended to be tolerated:

*“I had a case with chemical restraints that ended with an adverse-event report. Then I had a conversation with the nurse where we discussed it, and I understand her despair. At night there are so few staff; we have one health professional at each ward on 24 residents and one nurse responsible for all three wards with a total of 72 residents. It is scraped to the bone; there is no room for something to happen. Of course, it should not affect the resident, but at the same time, it is a problem that is not easy to solve in any way”.* (Care managers, group 2)

In contrast to the care managers perceptions, only one nursing home director mentioned inadequate staffing as a factor related to abuse. Instead, nursing home directors referred to the need for individual staff members to better prioritise their work and that a lack of correct prioritisation was a factor that could cause an incident. They remarked that they followed up on reports from staff regarding inadequate staffing by instructing care managers to help individual staff members to more effectively prioritise their work:

*“We have a budget that we need to have in balance. So, what I say to staff when they report low level of staffing is that, first of all, we have to ensure that we get the right things done in the right order. The staff need to know that they are allowed to prioritise. We had one case just now, where a staff member reported lack of time to follow up on the residents as an adverse event. I told the ward leader to tell the staff member that she can get help to prioritise her work, if she has a problem with that”*. (Nursing home directors, 8)

Although the care managers identified inadequate staffing as a factor related to abuse, they also indicated that they adjusted the service to the available resources and to align with norms in comparable nursing homes. They expressed that, when they received adverse-event reports from staff regarding neglect of resident care due to lack of time, they instructed staff regarding the available resources. However, if a resident showed aggressive behaviour, both the nursing home directors and care managers stated that they could put on extra staff. However, they indicated that it could be difficult to evaluate whether or not the extra staff was the appropriate solution.

#### Training and education

Participants described difficulty recruiting skilled staff and noted that many of the staff members at their nursing homes were unskilled. Both nursing home directors and care managers expressed concern that staff who lacked the necessary knowledge could increase the risk of resident abuse without being aware of it, and that unskilled staff may not be able to detect changes in residents’ health status, thereby risking neglecting a resident’s medical needs. At the same time, care managers said that they were unable to recruit adequately skilled staff, and, as a result, employing unskilled staff was necessary:

*“But, on the other hand, I don’t think we are able to do anything about this. We can’t manage without unskilled staff, and it is not going to happen that all unskilled personnel suddenly decide to become nurses (RNs) either, or that we can give a full position to every skilled nurse who wants it”.* (Care manager, group 4)

All participants said that they organised internal staff meetings with an educational focus on strategies to promote residents’ safety. Preventing the use of restraint and procedures related to its use were important topics for internal staff meetings. However, both nursing home directors and care managers stated that it was difficult to motivate and include all staff, especially night and weekend staff, in these meetings. Several participants stated that, to counterbalance these difficulties, their organisations employed a few staff members with extra training and knowledge related to restraint. These staff members had a special responsibility to guide and supervise other staff. Both nursing home directors and care managers said that they tried to encourage staff to guide each other and learn from others in the context of their everyday practice. They also pointed out the responsibility staff have for updating themselves on relevant knowledge. A nursing home director remarked: *“As professionals, the staff [members] have a duty to guide each other”* (Nursing home director, 12).

## Discussion

This study explored how nursing home leaders follow up on reports and information regarding staff-to-resident abuse. Nursing home directors and care managers described measures that were taken on an individual, group, and organisational level. An ambiguity emerged from the nursing home leaders’ examples of follow-up measures. On one hand, nursing home leaders indicated an intention to follow up on incidents of harm or distress to residents. On the other hand, they found it difficult to define harm stemming from abuse and felt powerless in terms of being able to follow up on all levels. An additional finding was that they lacked effective tools for evaluating the measures taken, and this influenced how and what leaders actually acted upon.

Participants in this study stated that they had little experience with reckless behaviour from staff, intentional physical acts, sexual abuse, or financial abuse. Even so, these incidents of evident abuse were perceived as incidents that should be acted upon by contacting the police and healthcare authorities. Intentional physical, sexual, and financial abuse do occur in nursing homes, but the frequency of these types of staff-to-resident abuse is low [13]. The most common forms of abuse are neglect and psychological abuse [11–13], but how the latter type is perceived influences what is reported. Hence, the incidents that nursing home leaders act on differ [19]. One important factor in determining abuse and its severity is the ability to consider a harmful situation from the perspective of the resident [7, 41, 42]. However, our study showed that when nursing home leaders investigated reports, they encountered varying and conflicting accounts of the incident. These accounts differed not only between staff members but also between residents and staff. It is concerning that leaders demonstrated a lack of confidence in the resident’s story, and this raises the question of whether their reactions are influenced by ageism. Certain behaviours such as abuse or discounting the stories of people with dementia seem to be justified and influenced by attitudes towards ageing in society [43]. This is also supported by previous research, indicating that abusive behaviour is rated as less serious when the resident has dementia [44].

Findings revealed that nursing home leaders in the present study linked incidents of abuse mainly to individual characteristics of the staff members involved, such as personality, attitude, personal problems, lack of knowledge, stress, and burnout. However, staff-to-resident abuse is a multifactorial problem [24, 25]. Understanding risks for staff-to-resident abuse in nursing homes requires a simultaneous focus on both the resident and the staff as a dyad and understanding the pattern of interaction that takes place between them within the contextual frame of the institution and the wider society [25]. There is a tendency in healthcare organisations to treat patient-safety issues as failings on the part of individual staff members [26, 45]. In contrast, a system-based approach focuses on the idea that most patient-safety issues reflect predictable human failings in the context of poorly designed systems [45, 46]. Nursing home leaders in our study wanted to identify the individual staff member involved in the situation in the adverse-event report so that they knew who to talk to when following up. According to international recommendations for national patient-safety incident-reporting systems, anonymous adverse-event reports are important because they prevent a ‘shaming and blaming culture’ [47]. Moreover, adverse-event reports should be collected for the purpose of learning rather than to address the failings of individual staff members [48]. Leaders play a central role in balanced accountability for both individual staff members and the organisation as a whole [26]. To determine whether the cause of an event is related to an individual’s reckless behaviour or to systemic factors, a systematic analysis approach is needed [26, 45, 49]. However, a systematic review of adverse-event analysis methods found that some approaches are limited because they do not capture the complexity of an adverse event [50]. This poses the question of whether the leaders in this study attempted to simplify the complexity of incidents of abuse by determining a linear solution of cause and effect and considering mainly individual factors rather than conducting a larger systematic analysis. It is alarming that some staff members were relocated as a follow-up when a leader became aware of their behaviour. Moving staff to another location where they continue to have the same care responsibilities will not facilitate individual or organisational learning.

The complexity of caring for residents with dementia and aggressive behaviour within a complex organisation was indicated by the participants to be a risk factor for abuse. Aggressive behaviour is complex and multifactorial, and can relate to individual resident factors, environmental factors and caregiver factors [51, 52]. Both staff’s and the organisation’s ability to meet residents’ needs and to adapt and cope with this complexity are challenged [51, 53]. According to complexity theory, people in complex systems will try to adapt to internal and external demands [5, 6]. This adaptation can have both positive and negative consequences [53]. Negative consequences of adaptation are seen when an abnormal culture becomes normal, for example, accepting the use of physical and chemical restraint, arguing with a resident, or rough handling during care [54]. One way leaders can facilitate learning within organisations is through feedback and openness [7, 29, 31]. Using adverse-event reports facilitates organisational learning and a just culture and avoids attributing blame to individuals [26]. Feedback and openness were also perceived as important by all participants, but how they implemented feedback from reports differed. Some leaders used adverse-event reports to promote organisational learning, even though most leaders gave feedback only to the specific staff member involved in an incident, which is a barrier for organisational learning. The leaders in this study stated that they often followed up on a group level by initiating reflective practices and, thereby, attempted to facilitate a cultural understanding of what constitutes abuse in the nursing home. Previous research has also found that, through reflection, long-held assumptions that form socially accepted behaviour within a culture can be challenged and changed by questioning existing processes and procedures [31, 32, 55]. This type of learning, developed by Argyris and Schön [31], is referred to as double-loop learning. The importance of systematic reflection has also been revealed in a Norwegian study, where staff caring for residents with aggressive behaviours enhanced their coping and learning skills through reflection [51]. However, the participants in our study stated that lack of time and resources was a main barrier to organisational learning through reflection. Many leaders responded to this barrier by placing the responsibility for reflection on individual staff members. However, the literature shows that organisational learning through reflection takes place when readiness to learn and a mental model for interpreting experiences are shared by staff [32]. This includes the idea that staff members must ‘learn how to learn’ with the use of reflection and must have time to do so [31, 32].

On an organisational level, findings from the present study suggest that care managers view inadequate staffing in regard to education and numbers as a contributing factor to staff-to-resident abuse. In contrast, nursing home directors stated that staffing was not the problem. Rather, the problem was related to incorrect prioritisation by individual staff members. Previous research found that lack of staffing in terms of education and numbers and high staff turnover were risk factors for abuse in nursing homes [12, 18, 21]. However, these studies did not include the perceptions of nursing home leaders. Care managers generally have less power than nursing home directors, which may result in a feeling of powerlessness to correct system defects. Care managers’ and nursing home directors’ differing perspectives about the association between staff and staff-to-resident abuse are noteworthy, particularly as previous research has identified associations between consistent leadership style in care managers and nursing home directors and quality of care and patient safety in nursing homes [27].

In order to meet nursing home residents’ needs for safe, high-quality care, a shared cultural understanding of the complexity of nursing home services and adequate staffing with the necessary competencies are required [9]. It is possible that nursing home directors respond to demands for efficiency and cost savings by putting the responsibility for preventing abuse onto staff members and attributing it to their individual prioritising instead of using their power to correct system defects. A further interesting finding is the lack of evaluation tools at a group and an organisational level. According to the national regulation of management and quality improvement in healthcare services in Norway, any follow-up of adverse events should also include an evaluation of the measures that were taken in response [33]. However, findings indicate that it is easier to identify changes in individual staff members’ behaviours than in the organisation or at the group level. This may explain the existence of a culture of blame in health care.

### Strengths and limitations of the study

This study involved participants from two levels of leadership from different nursing homes and municipalities in Norway, which is a strength and increases the transferability of the findings. Data collection methods consisted of both focus group interviews and individual interviews due to the difficulty of conducting focus group interviews with nursing home directors. Since nursing home directors and care managers can influence each other and jointly influence quality of care and patient safety, we viewed the advantages of including both data collection methods to be greater than the disadvantages since both methods are suitable for exploring people’s experiences with a specific phenomenon.

Three of the authors have worked as care managers in nursing homes for several years; this can be considered a strength as well as a limitation and requires a particular focus on reflexivity throughout the research process. Because of this background knowledge, it was possible to pose in-depth questions to explore a broad range of issues. However, background knowledge could also affect the type of follow-up questions asked during the interviews. To counterbalance this potential bias, two researchers were always present during the focus group interviews, and the analyses were coded by two researchers (JM and SN) independently. All findings were also discussed in the research group, which comprised researchers with broad research experience from two different countries. This, in turn, strengthens the trustworthiness of our findings and the credibility of the research.

## Conclusion

To prevent abuse of residents in nursing homes, it is important to understand how nursing home leaders follow up such incidents and what they follow up specifically. Our study revealed in-depth information about key factors related to how nursing home leaders react and act in response to elder abuse, which reflects their understanding of what constitutes abuse, its causal factors, and prevention strategies used in nursing homes. Nursing home leaders need to be clear about how they should follow up incidents of elder abuse on different levels in the organisation and their roles in its prevention. Nursing home leaders also need evaluation tools to facilitate systematic organisational learning. Moreover, nursing homes must operate as open, blame-free cultures that acknowledge that incidents of elder abuse in patient care arise not only from the actions of individuals but also from the complex everyday life of which they are a part and in which they operate.

## Data Availability

The datasets generated and/or analysed during the current study are not publicly available due to the format of the data not allowing for a complete anonymisation. However, data are available from the corresponding author on reasonable request.

## Abbreviations

NFR: Research Council of Norway
RN: Registered Nurse
WHO: World Health Organization
IHI: Institute for Healthcare Improvement
